# Sex Determination Using Inion-Opistocranium-Asterion (IOA) Triangle in Nigerians' Skulls

**DOI:** 10.1155/2014/747239

**Published:** 2014-05-18

**Authors:** C. N. Orish, B. C. Didia, H. B. Fawehinmi

**Affiliations:** Department of Anatomy, Faculty of Basic Medical Sciences, University of Port-Harcourt, PMB 5323, Choba, Port Harcourt, Rivers State, Nigeria

## Abstract

*Background*. Determination of sex is an important concern to the forensic anthropologists as it is critical for individual identification. This study has investigated the existence of sexual dimorphism in the dimensions and the area of the IOA triangle. * Methods*. A total of 100 adult dry skulls, (78 males; 22 females) from departments of anatomy in Nigerian universities were used for this study. Automatic digital calliper was used for the measurement. Coefficient of variation, correlation, linear regression, percentiles, and sexual dimorphism ratio were computed from the IOA triangle measurements. The IOA triangle area was compared between sexes. *Results*. The male parameters were significantly (*P* < 0.05) higher than female parameters. The left opistocranium-asterion length was 71.09 ± 0.56 and 61.68 ± 3.35 mm and the right opistocranium-asterion length was 69.73 ± 0.49 and 60.92 ± 2.10 mm for male and female, respectively. A total area of IOA triangle of 1938.88 mm^2^ and 1305.68 mm^2^ for male and female, respectively, was calculated. The left IOA indices were 46.42% and 37.40% in males and females, respectively, while the right IOA indices for males and females were 47.19% and 38.87%, respectively. * Conclusion*. The anthropometry of inion-opistocranium-asterion IOA triangle can be a guide in gender determination of unknown individuals.

## 1. Introduction

The existence of sexual dimorphism in human skeletons and its importance in medicolegal investigations have long been acknowledged. The skull is probably the most studied bone in that context. Krogman and Iscan [1] stated that sex assessment in a collection of 750 skeletons was possible, with levels of reliability of 100% when the entire skeleton was present, 92% using the skull alone, and 98% when combining the pelvis and skull. Even though several postcranial elements have more recently proved to be more effective sex predictors [2], the skull remains among the most dimorphic parts of the skeleton. Saavedra de Paiva and Segre [3] introduced an easy technique for sex determination starting from the temporal bone. The technique is based on the triangular area calculation obtained between these points: porion, mastoidale, and asterion, measured from xerographic copy of skulls. They found significant differences in the area between the right and left mastoid triangle when comparing male and female skulls, but owing to the asymmetries present in the skulls, it is recommended to observe the value of the total area (adding right and left sides), which was also significant, so that when it is higher than or equal to 1447.40 mm^2^, the skull is diagnosed as male skull, and a value near to 1260.36 mm^2^ or less is indicative of female skull [3]. Some of the earlier studies following this approach include those on Europeans [4, 5], Americans [6], South Africans [6–9], Japanese [10, 11], and Chinese [12] and had earlier researched on skull.

Despite the increase in research on sex prediction using craniofacial characteristics worldwide, information on such parameters is sparse in Nigerian population.

The aim of this study is to develop a sex determination technique using a triangle defined by these points: inion, opistocranium, and asterion; the union of these points determines the IOA triangle.

## 2. Materials and Method

A total of 100 adult dry skulls (78 males and 22 females), free from damage and deformity and fully ossified from departments of anatomy in Nigerian universities, were used for this study. A digital caliper with a precision of 0.01 mm (Mitutoyo) and marker were used to measure the following length parameters: opistocranium-asterion, opistocranium-inion, and inion-asterion. The skull was kept with Norma occipitalis facing the observer. 

*Inion*. Inion is the most prominent point in the posterior aspect of the occipital calvarium occurring at the intersection of the left and right superior nuchal lines. 

*Opistocranium*. Opistocranium is the most posteriorly protruding point on the back of the skull, located in the mid-sagittal plane. 

*Asterion*. Asterion is the meeting point of the lambdoid, occipitomastoid, and parietomastoid sutures or the point where the temporal, parietal, and occipital bones meet.

**Figure 1:**
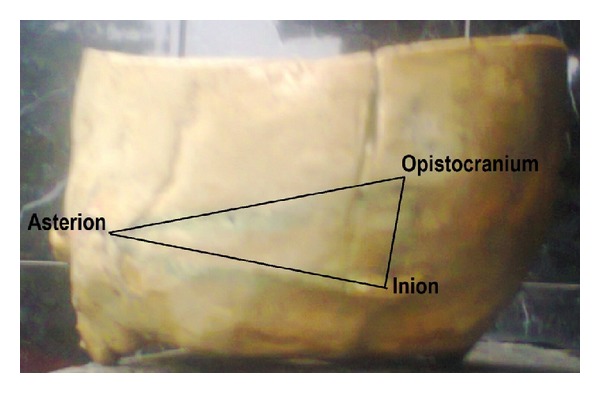
The area of the demarcated triangle as in Figure 1 was used for the results.

The union of these points determines the IOA triangle (Figure 1).

Data was anaylzed with Graph Pad Prism 5.03. The mean, standard deviation, standard error of mean, maximum, minimum, geometric mean, coefficient of variation, correlation, linear regression, percentiles, and sexual dimorphism ratio were computed. The IOA triangle area and IOA index were calculated and compared between males and females:

(1) $$\begin{array}{c} \text{IOA}\;\text{INDEX}=\frac{\text{Inion-opistocranium}}{\text{Inion-asterion}}\times 100. \end{array}$$

The results were shown in Tables 1 and 2 showing the measurements data of male and female skulls.  Table 3 shows the percentiles of right and left inion-opistocranium, right and left inion-asterion length, and the IOA index in males and females.  Table 4 shows the IOA index.  Table 5 shows the area of IOA triangle.  Table 6 shows sexual dimorphism ratio of the measurements inion-opistocranium, inion-asterion, and opistocranium-asterion.

The male/female ratios for the mean measurements were greater than unity, indicating that the male crania were larger in all linear dimensions than female crania. From the sexual dimorphism ratios calculated for recent Nigerian population inion-opisthocranion had the highest (1.34), while inion-asterion had the least (1.09) and opisthocranion-asterion had 1.15.

Correlation of craniometric parameters of Nigerian male and female populations is shown in Table 7. Male opistocranium-asterion left versus right had positive significant correlation, while inion-asterion left versus right had positive correlation. In the female population, opistocranium-asterion left versus right and inion-asterion left versus right had positive significant correlation.

**Figure 2:**
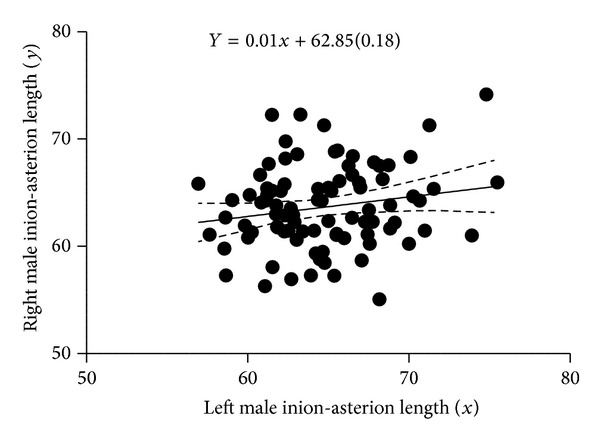
Linear regression graph of male left and right inion-asterion length.

**Figure 3:**
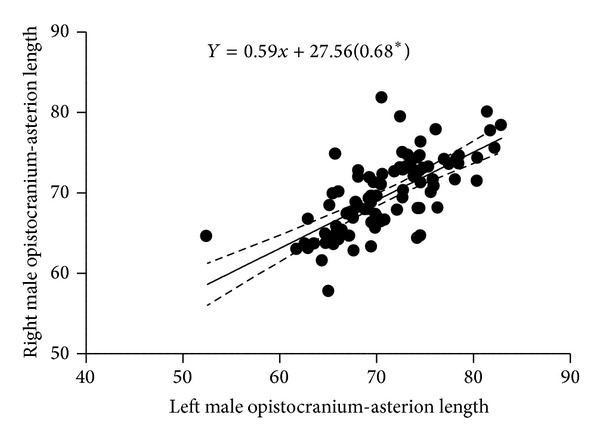
Linear regression graph of male left and right opistocranium-asterion length.

**Figure 4:**
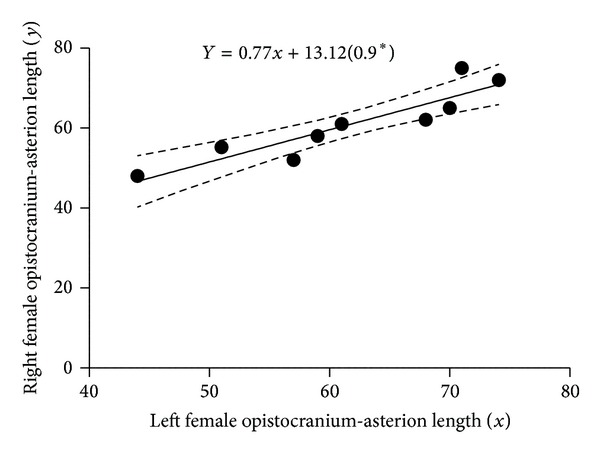
Linear regression graph of female and right opistocranium-asterion length.

**Figure 5:**
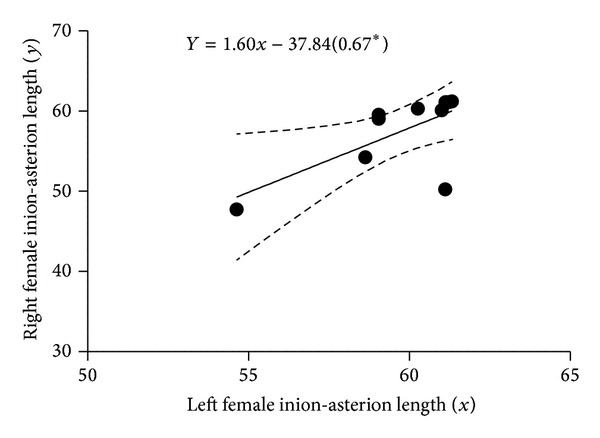
Linear regression graph of female left and right inion-asterion length.

Figure 2 is a scatter plot of the linear relationship between left and right male inion-asterion length. There was positive correlation between the left and right; hence, the fit line sloped upward.  Figure 3 shows a scatter plot of the linear relationship between left and right male opistocranium-asterion. There was positive correlation between the left and right; hence, the fit line sloped upward.  Figure 4 shows a scatter plot of the linear relationship between left and right female opistocranium-asterion. There was positive correlation between the left and right; hence, the fit line sloped upward.  Figure 5 shows a scatter plot of the linear relationship between left and right female inion-asterion length. There was positive correlation between the left and right; hence, the fit line sloped upward.

## 3. Discussion

Accurate determination of sex from the human skull is of great importance to osteologists and the forensic anthropologists as it is critical for individual identification. It eliminates approximately 50% of the population from further consideration in cases of missing persons. Moreover, many additional individualization criteria are sex specific [1–13]. The morphological differences between both sexes can be the result of multiple factors such as genetic factors, for example, a relative fixed racial genome, but the phenotypic expression is modified by multiple factors such as local customs and environmental factors affecting growth and development (nutrition, physical activity, life-style, health, etc.). The only constants in this complex equation are the biological sex controlled by sex chromosomes and genetic and/or racial heritage [1, 14, 15]. Studies on sexual dimorphism are based on three primary biological differences between males and females, which are size, body proportions, and architectural differences [16]. Males are generally more robust than females as they have generally more muscle mass. The weight of the axial skeleton of the male is relatively and absolutely heavier than that of the female by about 8% [16].

This might be the first report on sex determination technique using IOA triangle as we are not aware of any previous data. However, in other related studies, [8, 17] showed that male parameters were higher than female and statistically significant at *P* < 0.05 which correlates well with the present findings. The length of male inion-opistocranium was significantly different from the female. The length of left and right male inion-asterion was significantly higher than that of the female. There was a significant difference between male left and right opistocranium-asterion from female.

Area of IOA triangle of male was found to be significantly higher than that of female. Although there seems to be no previous report on this parameter, our result on IOA triangle seems to agree with the findings of [3, 18] on mastoid triangle of Caucasians skulls using Heron's report that included that the total area is higher in male than in female.

Ahmed et al. [19] reported that sexual dimorphism ratio (male/female ratios) for the mean measurements was greater than unity, indicating that the male crania were larger in all linear dimensions than female crania. From the sexual dimorphism ratios calculated for Sudanese population, the ratios were Basion-prosthion, and Basion-bregma (1.05), Bregma-lambda (1.02), Basion-nasion (1.04), Basion-prosthion (1.06), and Nasion-bregma (1.04). These data are similar to the present study that has presented sexual dimorphism ratio is greater than unity, indicating that the male crania were larger in all linear dimensions than female crania.

Hitherto information is scanty on the mathematical models of these craniometric parameters [20]. A positive regression coefficient indicates a positive relationship between two variables and from the graph the fit line sloped upward as in male and female opistocranium-asterion left versus right and inion-asterion left versus right. The present result showed that male opistocranium-asterion left versus right had positive significant correlation, while inion-asterion left versus right had positive correlation, whereas female opistocranium-asterion left versus right and inion-asterion left versus right had positive significant correlation. This study has also documented that the percentiles of right and left inion-opistocranium, right and left inion-asterion length left IOA index progressively increased in values from 10th percentiles to 90th in both sexes with male values higher than female values except female right IOA index which showed a higher value than that of male. The male IOA index was higher than that of female.

Taken together the present research showed high level of sexual dimorphism and will be of immense help to forensic expert. Further research will validate these findings.

## Figures and Tables

**Table 1:** Maximum, minimum, geometric mean, coefficient of variation, mean, and SEM of inion-opistocranium length, left inion-asterion length (bracket)^#^,and right inion-asterion length [parentheses]^∞^ (mm).

Sex	Mean ± SEM	Coefficient of variation	Geometric mean	Maximum	Minimum
Male (mm)	30.03 ± 0.50	16.01%	29.67	46.59	22.21
	(64.69 ± 0.40)^#^	(5.91%)^#^	(64.58)^#^	(75.49)^#^	(56.94)^#^
	[63.64 ± 0.40]^*∞*^	[6.03%]^∞^	[63.53]^∞^	[74.17]^∞^	[55.06]^∞^
Female (mm)	22.34 ± 2.10*	29.68%	21.47	32.22	14.10
	(59.74 ± 0.65*)^#^	(3.47%)^#^	(59.70)^#^	(61.32)^#^	(54.63)^#^
	[57.48 ± 1.56*]^*∞*^	[8.59%]^∞^	[57.27]^∞^	[61.22]^∞^	[47.76]^∞^

*P < 0.05 statistically significant from male.

Numbers in brackets represent left inion-asterion length^#^ and numbers in parentheses represent right inion-asterion length [parentheses]^∞^ (mm).

**Table 2:** Maximum, minimum, geometric mean, coefficient of variation, mean, and SEM of left opistocranium-asterion length and right opistocranium-asterion length (mm).

Sex	Mean ± SEM	Coefficient of variation	Geometric mean	Maximum	Minimum
Male (mm)	71.09 ± 0.56	7.50%	70.88	82.95	52.51
	(69.73 ± 0.49)^#^	(6.67%)	(69.58)	(81.79)	(57.71)
Female (mm)	61.68 ± 3.35*	16.28%	60.90	74.10	44.00
	(60.92 ± 2.10*)^#^	(14.53%)	(60.35)	(75.00)	(48.00)

*P < 0.05 statistically significant. Numbers in brackets represent right opistocranium-asterion length (mm)^#^.

**Table 3:** Percentiles of inion-opistocranium, right and left^#^ inion-asterion length and right and left^#^ IOA index in males and females.

	Sex	10th	25th	50th (Median)	75th	90th	Normality test
Inion-opistocranium length	Male	24.43	26.66	29.83	32.56	34.88	<0.0001*
	Female	14.21	15.83	21.16	26.67	32.10	0.4179

Inion-asterion length	Male	58.71	61.11	63.39	65.93	68.54	0.4629
		[60.15]^#^	[61.83]	[64.48]	[67.27]	[70.09]	[0.1111]
	Female	48.01	53.25	59.84	61.11	61.21	0.1598
		[55.03]^#^	[58.95]	[60.63]	[61.14]	[61.31]	[0.0027*]

Right/[Left]^#^ IOA index	Male	39.24	41.95	46.06	52.07	56.46	0.0002
		[36.22]^#^	[41.54]	[45.52]	[50.32]	[56.66]	[0.0001*]

*P < 0.05 statistically significant from male.

^#^Data in parenthese represent Percentiles of left length, inion-opistocranium, and left inion-asterion; left IOA index.

**Table 4:** IOA index.

Sex	Left IOA index (%)	Right IOA index (%)	Average IOA index (%)
Male	46.42	47.19	46.81
Female	37.40	38.87	38.14

**Table 5:** Area of IOA triangle mm^2^.

	Left	Right	Total area
Male	972.17	966.71	1938.88
Female	664.04*	641.64*	1305.68*

*P < 0.05 statistically significant from male.

**Table 6:** Sexual dimorphism ratio.

Parameters	Male mean (mm)	Female mean (mm)	Sexual dimorphism ratio = M. mean/F. mean
Inion-opistocranium	30.03	22.34	1.34
Inion-asterion	64.16	58.61	1.09
Opistocranium-asterion	70.41	61.30	1.15

**Table 7:** Correlation of craniometric parameters of Nigerian male and female populations.

Male craniometric parameter (*x* versus *y*)	Regression (correlation, *r*)
Opistocranium-asterion left versus right	*Y* = 0.59*x* + 27.56 (0.68*)
Inion-asterion left versus right	*Y* = 0.01*x* + 62.85 (0.18)
Female craniometric parameter (*x* versus *y*)	Regression (correlation, *r*)
Opistocranium-asterion left versus right	*Y* = 0.77*x* + 13.12 (0.9*)
Inion-asterion left versus right	*Y* = 1.601*x* − 37.84 (0.67*)

*Stastical significance.
